# Opuntia in México: Identifying Priority Areas for Conserving Biodiversity in a Multi-Use Landscape

**DOI:** 10.1371/journal.pone.0036650

**Published:** 2012-05-14

**Authors:** Patricia Illoldi-Rangel, Michael Ciarleglio, Leia Sheinvar, Miguel Linaje, Victor Sánchez-Cordero, Sahotra Sarkar

**Affiliations:** 1 Biodiversity and Biocultural Conservation Laboratory, Section of Integrative Biology, University of Texas, Austin, Texas, United States of America; 2 Jardín Botánico Exterior, Instituto de Biología, Universidad Nacional Autónoma de México, Coyoaccán, México D.F.; 3 Laboratorio de Sistemas de Información Geográfica, Departamento de Zoología, Instituto de Biología, Universidad Nacional Autónoma de México, Circuito Exterior, Coyoacán, México D.F.; 4 Department of Philosophy, University of Texas, Austin, Texas, United States of America; University of Western Ontario, Canada

## Abstract

**Background:**

México is one of the world's centers of species diversity (richness) for *Opuntia* cacti. Yet, in spite of their economic and ecological importance, *Opuntia* species remain poorly studied and protected in México. Many of the species are sparsely but widely distributed across the landscape and are subject to a variety of human uses, so devising implementable conservation plans for them presents formidable difficulties. Multi–criteria analysis can be used to design a spatially coherent conservation area network while permitting sustainable human usage.

**Methods and Findings:**

Species distribution models were created for 60 *Opuntia* species using MaxEnt. Targets of representation within conservation area networks were assigned at 100% for the geographically rarest species and 10% for the most common ones. Three different conservation plans were developed to represent the species within these networks using total area, shape, and connectivity as relevant criteria. Multi–criteria analysis and a metaheuristic adaptive tabu search algorithm were used to search for optimal solutions. The plans were built on the existing protected areas of México and prioritized additional areas for management for the persistence of *Opuntia* species. All plans required around one–third of México's total area to be prioritized for attention for *Opuntia* conservation, underscoring the implausibility of *Opuntia* conservation through traditional land reservation. Tabu search turned out to be both computationally tractable and easily implementable for search problems of this kind.

**Conclusions:**

*Opuntia* conservation in México require the management of large areas of land for multiple uses. The multi-criteria analyses identified priority areas and organized them in large contiguous blocks that can be effectively managed. A high level of connectivity was established among the prioritized areas resulting in the enhancement of possible modes of plant dispersal as well as only a small number of blocks that would be recommended for conservation management.

## Introduction

Traditional systematic conservation planning for biodiversity consists of selecting priority areas to be designated as strictly protected areas such as national parks or, more recently, as reserves that permit some human; nevertheless, a conceptual difference exists between areas that are protected and those that are not [1]–[3]. However, if the biota of interest (the biodiversity “surrogates” *sensu* Sarkar and Margules [4]) is dispersed at low densities over extended landscapes (for instance, on a continental scale) that provide livelihoods for human populations, designating such protected areas is typically neither feasible nor appropriate. The maintenance of viable populations of all species would require far too large an area that would have to be set aside from continued human use. At the pragmatic level, any such policy is likely to fail because successful conservation requires local support, which would possibly not be forthcoming if livelihoods were threatened [5], [6]. More importantly, any policy that seriously threatens (human) livelihoods only in the interest of preserving biota is not ethically defensible [5], [7], [8]. This problem is further intensified, especially in societies with economically disadvantaged communities, when the biota to be protected themselves also have everyday tangible human use, for instance, as food or construction materials. It is imperative in such situations to find alternative management strategies for conservation that go beyond the protected areas model.

What is required in such circumstances is a reconceptualization of priority areas so that they are not necessarily thought of as areas qualitatively different from those that are in everyday human use in the surrounding landscape matrix. These priority areas are still conservation areas (*sensu* Sarkar [9]) but conservation means that management is designed to foster the persistence of biodiversity components: there is no endorsement, explicit or implicit, that the management mode requires human exclusion and is thus akin to the traditional national park model. This situation requires that traditional conservation area selection methods [10] be modified to take into account a variety of factors beyond the traditional ecological ones of ensuring the adequate representation and persistence of biodiversity [1]. The problem presents both constraints and opportunities. With respect to the spatial organization of priority areas, the constraints can be operationalized as three criteria ideally to be satisfied: (i) areas should be compact for ease of management; (ii) they should be connected, so that there are as few distinct management units as possible; and (iii) preferably, the areas should be aligned to a well-defined ecosystem (habitat) type, or to existing protected areas, or to politically homogeneous spatial units, so that a single management strategy (or a small number of them) is adequate. (Some of these constraints are also desirable in many circumstances to encourage the persistence of species, for instance, dispersal [2], [10] but that issue will remain in the background in this paper). The major opportunity is that the size (area) of the network of priority areas need not be the absolute minimum to represent the biodiversity surrogates. Since the goal is to foster the persistence of the biodiversity while allowing human use, there can be tradeoffs between spatial coherence and area minimization. If conservation areas are to remain in human use, larger areas can be designated for conservation than if they were to be precluded from such use. Socio-economic criteria can be incorporated into algorithms for the prioritization of individual conservation areas. Alternatively, sets of conservation area networks may be algorithmically identify on the basis of biodiversity representation and management concerns, as discussed earlier, and further socio-economic considerations then used to select one of these sets. The distinction here is between iterative and terminal stage selection, respectively, of conservation area networks [5], [11]: in iterative protocols, all socio-economic criteria are incorporated into algorithms as each area is included in a set of nominal conservation areas; in terminal stage protocols, entire sets of conservation areas are also assayed for their performance under the socio-economic criteria. For both protocols, multi-criteria analysis (MCA) must be used to incorporate the various biological, spatial, and socio-economic factors [10]. If the study region (that is, the region for which the analysis is being performed) is large, and the resolution used is fine (in this case 0.02°), then precise socio-economic data are typically not available for each potential habitat unit; this is the case for the study area considered here. In such contexts, an iterative selection of conservation areas incorporating all criteria is not possible and a terminal stage protocol must be used.

Within the context of a terminal stage protocol for *Opuntia* conservation throughout México, this analysis prioritizes sets of areas (nominal conservation area networks) that satisfy the biodiversity representation and spatial management criteria using a comprehensive data set assembled over the last 20 years. This is done at a resolution that is fine enough for policy formulation at the local level of municipalities, which are the most relevant entities in México for devising policies for the persistence of these species while maintaining their human use. The size of the data set presents formidable computational problems. If issues of spatial coherence (compactness, connectivity, *etc*.) are ignored, the formal (mathematical and computational) problem of selecting conservation areas is well–studied [10], [12], [13], and even large problems can now be solved using optimal algorithms, that is, those that provide an exact solution to the optimization problem. However, optimal algorithms have yet to be devised to solve complex spatial problems in reasonable times [10]. Heuristic algorithms, which are supposed to produce approximately best solutions, are known not to solve spatial problems adequately. Consequently, the preferred alternative is to use metaheuristic algorithms. (This terminology and the relevant issues will be explained in detail in the Materials and Methods section). In the past, simulated annealing has often been used for this purpose, especially as incorporated in the Marxan software package [14]. However, it has been found to be relatively slow [15], and Marxan only permits the use of a pre-specified set of criteria (size, shape [compactness], and a generalized cost) [16]. This analysis uses a tabu search algorithm [17] and a new (soon to be released) version of the ConsNet software package [18], [19]. Tabu search is known to be a fast metaheuristic algorithm for a wide variety of optimization problems [17]. Besides implementing tabu search, ConsNet allows the incorporation of an indefinite number of criteria into the identification of priority sets using a modification of the Analytic Hierarchy Process (AHP) [20], which ensures that the multi-criteria analysis is consistent with multi-attribute value theory (MAVT) [21], [22]. ConsNet has inbuilt algorithms to incorporate compactness and connectivity. Other criteria can be modeled using input from the user.

The group *Opuntia* (prickly pear or nopal) consists of two genera, *Opuntia* and *Nopalea*, of the Cactaceae family. The group evolutionarily originated in the American continent, and species from these genera can be found from just south of the Arctic circle in Canada to the tip of Patagonia in South America [23], and from sea level to an altitude of 5 100 m in Peru [24], in climates with no more than 500 cm of annual precipitation [23]. The country is the world's most important center of diversity of genera and species of cacti, including *Opuntia*, most of which are endemic to it (73% and 78%, respectively, for genera and species [25]). There exist about 200 recognized species, of which at least 84 are found in México (and, depending on taxonomic choices, these numbers may be higher) [26]. México thus has one of the world's highest *Opuntia* species richness [27], so conservation of this genera is important for both biodiversity persistence and economic sustainability. Most of these species occur in arid or semiarid regions, where they are subject to different types of threat generally due to human activities, primarily habitat conversion, but also unsustainable harvesting for direct use and for sale in national and international markets [25], [28]. These sales are economically important because these cacti are often ideal crops for arid regimes [23]. However, because of the threats, most genera are legally recognized as being in need of a certain level of protection.

Two factors mitigate against designating strictly protected areas for *Opuntia* conservation in Mexico. First, there is extensive endemism, including micro endemism (see Table 1), but the endemics are widely dispersed over the arid regions of the country. Consequently, conservation cannot be focused on a small set of specific areas to be legally designated as strictly protected and removed from routine human use. Second, in their native habitat, *Opuntia* species have extensive human use: to feed cattle, goats, sheep, and horses, and to prepare food and other derived products for human consumption while they are also a food source for a variety of wild fauna [25], [29]. Such use has resulted in two developments that have conservation implications: (i) an increase in hybridization between species that were brought under domestication from wild populations; and (ii) a decrease in morphological diversity within isolated domesticated populations which may eventually lead to a decrease in genetic variation [30]. The first development leads to the problematic situation that *Opuntia* taxonomy remains in flux; consequently, conservation goals should include large areas for management so that all taxa of potential value are likely to be represented (which may not happen if there is a focus on a small “optimal” set of areas). The second development also entails the same recommendation but for the goal of maintaining as much genetic diversity as possible.

**Table 1:** *Opuntia* Species in México.

Species	No. of Records	Status	AUC	Total Representation	Target (%)	
*O. albicarpa*	15	E	0.97	9820.17	76.44	
*O. amarilla*	6	E	0.99	2957.95	93.22	
*O. andersonii*	11	ME	1	793.83	98.64	
*O. atrispina*	4	E	1	912.38	98.32	
*O. atropes*	155	E	0.97	8389.94	80.31	
*O. azurea*	12	ME	0.94	16487.34	60.45	
*O. basilaris*	1	E			0	
*O. bensonii*	18	E	0.99	11563.69	72.03	
*O. bravoana*	3	ME			0	
*O. chaffeyi*	4	ME	1	176.35	100	
*O. chavena*	81	E	0.99	8497.89	79.94	
*O. chlorotica*	17	ME	0.97	33807.62	15.19	
*O. cochinera*	41	E	0.98	3045.79	92.94	
*O. cretochaeta*	8	E	0.94	34174.75	10	
*O. decumbens*	65	NE	0.92	10869.89	10	
*O. depressa*	46	E	0.98	5183.09	88.54	
*O. durangensis*	46	E	0.99	8115.1	80.64	
*O. elizondoana*	6	ME	1	3922.38	91.04	
*O. engelmannii*	541	E	0.94	11484.34	73.64	
*O. erinacea*	2	E			0	
*O. excelsa*	47	E	0.99	3060.88	93.51	
*O. feroacantha*	9	ME	0.97	5595.17	86.02	
*O. fragilis*	1	ME			0	
*O. fuliginosa*	206	E	0.95	10672.4	74.61	
*O. grahamii*	1	ME			0	
*O. guilanchi*	43	E	0.96	15867.8	60.05	
*O. heliabravoana*	39	E	0.98	1432.24	97.24	
*O. howeyi*	2	ME			0	
*O. huajuapensis*	25	E	0.99	3621.17	92.11	
*O. humifusa*	1	ME			0	
*O. hyptiacantha*	144	E	0.97	7069.52	82.85	
*O. icterica*	220	E	0.96	9404.29	76.82	
*O. incarnadilla*	12	E	1	3695.09	91.31	
*O. joconostle*	104	E	0.97	7516.44	82.24	
*O. lasiacantha*	158	E	0.95	9717.48	76.15	
*O. leucotricha*	132	E	0.97	6919.58	83.67	
*O. littoralis*	10	E	0.6	14805.21	0	
*O. macrocentra*	7	ME	0.96	10434.75	74.06	
*O. macrorhiza*	9	E	0.95	18508.28	55.65	
*O. matudae*	30	E	0.99	3092.02	92.98	
*O. megacantha*	72	E	0.96	8038.74	79.24	
*O. megarhiza*	16	E	1	3563.6	91.42	
*O. microdasys*	163	E	0.94	9665.43	77.96	
*O. nejapensis*	2	E			0	
*O. neochrysacantha*	1	ME			0	
*O. nigrita*	13	E	0.9	25834.49	32.5	
*O. oligacantha*	24	E	0.97	7617.36	82	
*O. olmeca*	1	ME			0	
*O. orbiculata*	38	E			0	
*O. oricola*	2	ME			0	
*O. pachona*	11	E	0.96	10811.65	72.59	
*O. pachyrhiza*	7	E	0.99	9949.49	76.7	
*O. parviclada*	102	ME	0.98	14158.64	66.46	
*O. phaeacantha*	66	E	0.82	28255.9	0	
*O. pilifera*	1	E		3288.14	92.62	
*O. polyacantha*	2	ME			0	
*O. pottsii*	166	ME			0	
*O. puberula*	79	NE	0.97	8844.52	10	
*O. pubescens*	49	E	0.95	12250.87	70.68	
*O. pumila*	4	E	0.97	6144.97	86.51	
*O. pycnacantha*	2	E			0	
*O. pyriformis*	1	E			0	
*O. reflexispina*	11	E			0	
*O. rileyi*	265	E	0.96	21695.89	49.48	
*O. ritteri*	1	ME			0	
*O. robusta*	28	E	0.94	9718.18	76.72	
*O. rzendowskii*	7	E	1	1343.62	97.37	
*O. scheeri*	2	ME	0.22	2218.24	0	
*O. schotti*	2	E			0	
*O. setispina*	12	ME			0	
*O. spinulifera*	11	E	0.95	8732.19	78.05	
*O. spraguei*	247	E	0.96	26206.42	38.59	
*O. stenopetala*	218	E	0.98	4791.66	88.43	
*O. streptacantha*	115	E	0.97	8009.59	81.31	
*O. stricta*	7	NE	0.94	18941.48	10	
*O. tapona*	5	E	0.96	7270.1	82.12	
*O. tehuacana*	204	ME	1	30053.72	25.37	
*O. tomentosa*	16	E	0.97	8091.83	80.84	
*O. undulata*	81	E	0.92	32890.73	14.02	
*O. velutina*	4	E	0.96	7921.01	81.29	
*O. vilis*	68	ME	0.98	7039.74	83.72	
*O. violacea*	21	E	0.73	41333.63	0	
*O. wilcoxii*	16	E	0.84	19920.63	53.65	
*O. zamudioi*	11	E	0.99	21133.86	48.44	

All 84 *Opuntia* species from México are included. AUC values have been rounded off to two decimal places. The absence of an AUC value indicates that no species distribution model was constructed. The absence of a target indicates that the model did not satisfy the adequacy criteria for use in the prioritization exercise. The targets are percentages of the total expected value for the species in the study area. With respect to status: E  =  endemic; ME  = microendemic (rare); NE  =  non–endemic.

Where they occur, *Opuntia* species are locally abundant and often form dominant components of natural floras, especially in arid regions, where they have substantial environmental importance. They are a major ecological component of the floras of the Chihuahuan and Sonoran Deserts (where they form assemblages also known as nopaleras). They contribute significantly to soil stability and constitute an important dietary component of a variety of mammal species including white-tailed and mule deer (*Odoicoileus virginianus* and *O. hemionus*), rodents (*Peromyscus*, *Dipodomys*, and *Neotoma spp*.), and coyotes (*Canis latrans*) [31], [32]. They also provide nesting sites and food for a variety of insects, birds, rodents, and lagomorphs [30]. In spite of their economic and environmental importance, *Opuntia* species remain relatively little studied [24], and a systematic conservation plan for them has not previously been formulated for México (or elsewhere).

The purpose of this study is to develop such a systematic plan by prioritizing areas for conservation attention. We addressed the hypotheses that an adequate *Opuntia* conservation in México (i) require a much larger area than could reasonably be put under protection without routine human presence and use; (ii) require incorporation of a wide variety of criteria partly to make management feasible; and (iii) require innovative computational algorithms to find satisfactory solutions that have a significant potential for implementation on the ground. The methodology developed here was specifically applied to Mexico but can be transported with no modification to other areas which are centers of *Opuntia* diversity. Moreover, it is equally applicable to all widely-dispersed taxa. First, a standard maximum entropy algorithm [33], [34] was used to create species distribution models for 60 *Opuntia* species based on occurrence data and environmental variables. Second, targets of representation were assigned to *Opuntia* species which were inversely proportional to their estimated ranges in order to prioritize endemic and other rare species. Third, the area prioritization problem was reformulated as a constrained optimization problem. There were two hard constraints: the satisfaction of the biodiversity representation targets just mentioned, and the inclusion of all existing formally protected areas as priority areas. The latter constraint incorporated the spatial criterion of alignment to the extent required by this problem. The other spatial criteria, which are related to management options, were incorporated into an objective function to be minimized with weights on the area, connectivity, and shape of priority areas. Minimization was achieved using a modular adaptive tabu search algorithm [35]. Three different objective functions were used and each resulted in a map of the distribution of priority areas for *Opuntia* conservation in México. These three plans are thus available to policy-makers to be ranked on the basis of socio-economic and other criteria. Beyond the identification of sets of priority areas, the analysis here does not aim to devise management plans because these will depend on detailed analyses of local contextual preferences after a final set of priority areas have been selected.

## Results and Discussion

### Species' Distribution Models and Targets

**Figure 1:**
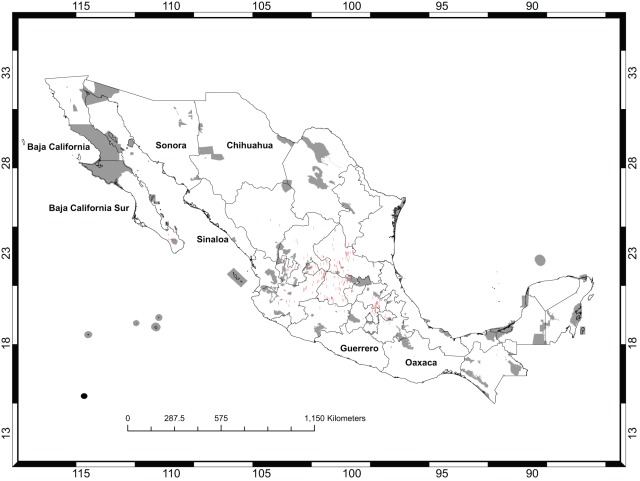
*Opuntia* Records in México. The existing protected areas are shown in gray. The red dots show the sites from which *Opuntia* occurrence records were available. The states that are named are those that are mentioned later in the Discussion.

**Figure 2:**
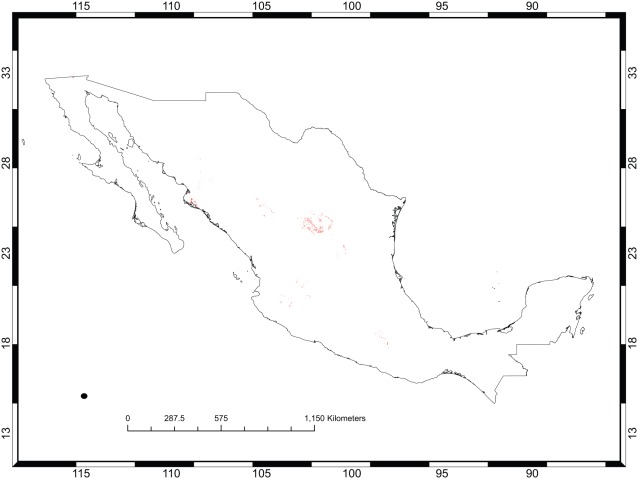
The predicted distribution of *Opuntia chaffeyi* in México. This distribution model was created using only four records and, accordingly, shows a highly restricted range. Darker areas have higher predicted habitat suitability.

**Figure 3.Distribution:**
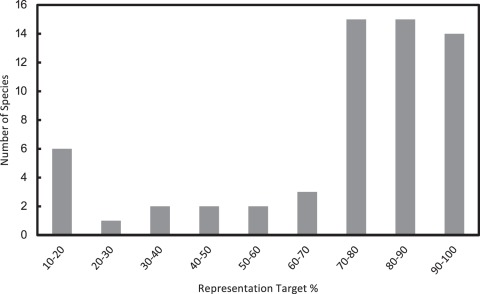
Distribution of representation targets. A large number of species have high percentage targets indicating highly restricted ranges.

Figure 1 shows the occurrence records for *Opuntia* in México and the existing protected areas. Models for 60 species satisfied the adequacy criteria used for this analysis (Table 1), that is, they were deemed accurate enough to be used to prioritize areas for conservation management. Figure 2 shows the predicted distribution for *O. chaffeyi*, which had the fewest occurrence records (four data points). This species exhibits an extreme form of micro endemism, which was quite common for this dataset (see Figure 3). Models for three species had AUC values <0.8 and were rejected: *O. littoralis*, *O. phaeacantha*, and *O. violacea*; these had 10, 66, and 21 records, respectively.

Figure 3 shows the distribution of the number of species versus their percentage targets of representation. Because there were many species with a low total representation (Table 1), that is the number of cells in which they are expected to occur, a large number of the species have very high percentage targets. Even though, at least for microendemics, this high percentage need not translate into large areas, this result is consistent with the presumed difficulty of attempts at *Opuntia* conservation through the creation of strictly protected areas.

### Conservation Area Networks

There were 39 475 cells designated as protected areas at the resolution of this analysis. This means that about 9% of the total area of México is formally protected. Given that, globally, setting aside 10% or 12% of the area of each country for biodiversity protection is usually regarded as a sufficiently ambitious goal [10], it would be socio-politically difficult to designate more areas for strict protection for *Opuntia* conservation.

**Figure 4:**
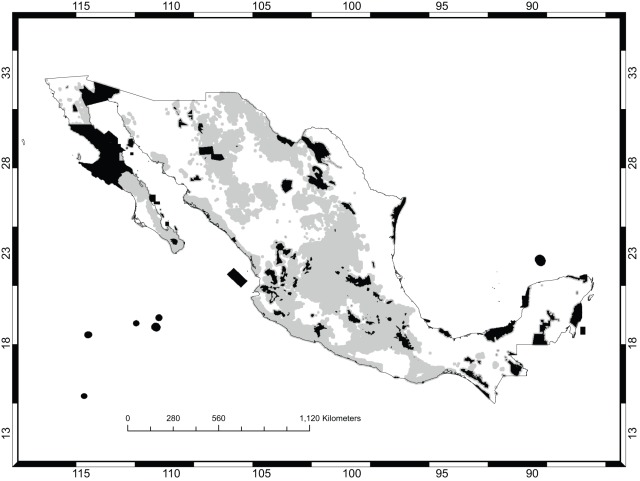
Plan A. The existing protected areas are shown in black. The additional selected areas are in gray.

**Figure 5:**
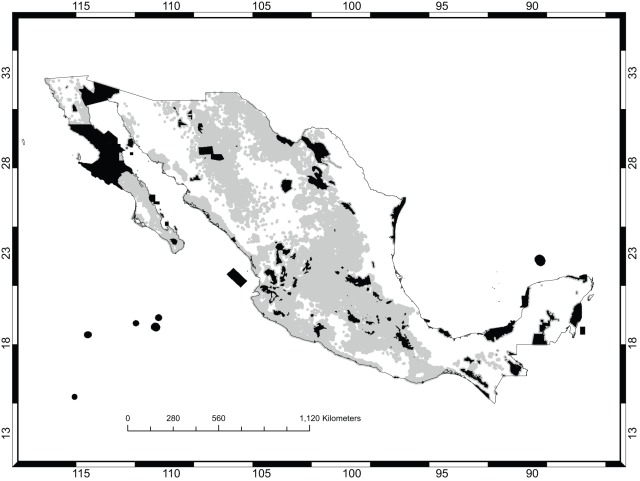
Plan B. The existing protected areas are shown in black. The additional selected areas are in gray.

**Figure 6:**
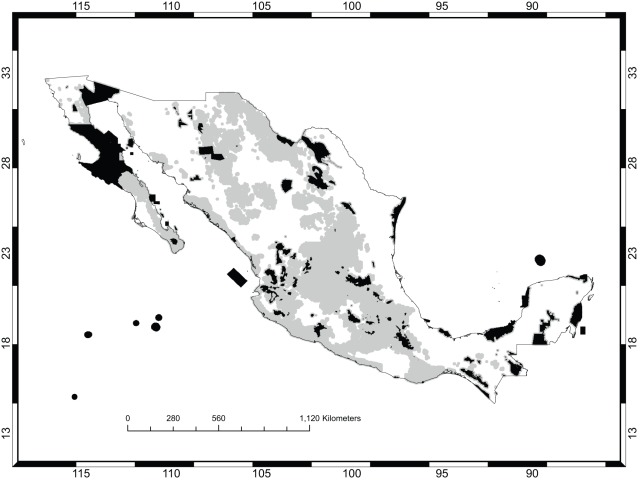
Plan C. The existing protected areas are shown in black. The additional selected areas are in gray.

**Figure 7:**
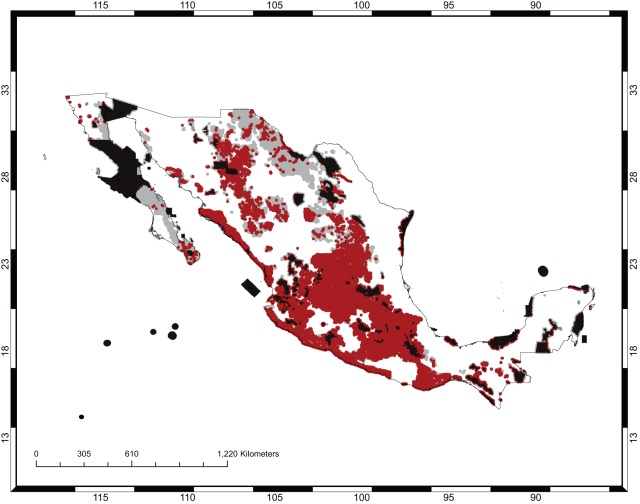
Agricultural and forestry areas in the new prioritized cells under Plan C1. These are shown in red when they intersect with the new prioritized areas (that is, other than those in the existing protected areas). The solution corresponds to that in Figure 6.

If no spatial criteria were used (the “null” solution in the discussion below), all targets of representation for all species can be achieved in 133 570 new cells, that is, cells outside the existing protected areas. Such a solution has 10 221 clusters (or connected components). When spatial criteria were used, three separate nominal conservation plans were formulated. Plan A incorporated the minimization of area and maximization of compactness with equal weights. Plan B gave a three-fold preference to the former. Plan C included these criteria with equal weights but also incorporated achieving connectivity with a relative weight of one-half compared to the other two criteria. For each plan, two different solutions (labeled“1” and “2”) were obtained using different starting points for the search. Figure 4 shows the solution or conservation area network selected under Plan A with the least area; Figure 5 is the corresponding map for Plan B. Figure 6 is the map with highest connectivity for Plan C. Table 2 gives the number of cells, shape value (the perimeter–to–area ratio), and the number of clusters. Finally, all plans require about one-third of México's total area to be put under conservation. Table 3 shows the extent to which the major vegetation types of México were included in the different plans. It did not come as a surprise that the dominant vegetation type, under all plans, was xeric scrubland since these are assemblages typically dominated by *Opuntia* species. More pertinent to this study was the result that the next most common vegetation type consisted of agricultural and forestry lands. Since these are subject to intensive human use, their prevalence underscores the point being emphasized here, that *Opuntia* conservation areas should not be conceptualized as regions of strict human exclosure. Rather, both strictly protected areas for the conservation of microendemics, and management programs admitting human use for widely distributed species should be devised and should focus only on the long-term persistence of *Opuntia* species. For example, Figure 7 shows the agricultural and forestry lands incorporated into Plan C1. All plans included substantial areas of oak, pine, and deciduous forests, which were not intuitively expected until the performance of this analysis.

**Table 2:** Spatial properties of Plans.

Plan	No. of	Perimeter–area	No. of
	New Cells	Ratio	Clusters
A1	153 478	0.098	598
A2	153 694	0.098	659
B1	140 081	0.207	2 317
B2	140 636	0.205	2 089
C1	153 941	0.097	265
C2	152 461	0.11	316
Null	133 570	0.416	10 221

A, B, and C refers to the three plans; 1 refers to the first solution; 2 to the second. The Null plan is the one in which no spatial criteria are used.

**Table 3:** Vegetational analysis of plans.

Vegetation Type	Null	A1	A2	B1	B2	C1	C2
Xeric Scrubland	201557	229 351	231 244	205 868	208 568	224 186	229 940
Agricultural–Forestry Lands	158660	178 371	173 712	176 023	176 236	186 449	179 503
Oak Forest	50209	57 207	64 649	52 311	53 685	56 116	60 190
Coniferous Forest	49207	67 860	66 088	53 560	52 715	68 565	63 401
Deciduous Forest	44177	51 606	50 344	45 662	45 899	50 743	48 210
Grassland	31663	39 626	36 967	30 411	31 023	39 333	36 818
Rainforest	14890	15 382	15 066	15 335	14 222	15 460	14 894
Aquatic Vegetation	13442	13 771	13 618	13 275	13 224	13 567	13 591
Sub-deciduous Forest	7224	8 208	8 853	8 180	8 055	8 083	8 301
Cloud Forest	5628	6 106	6 222	6 612	5 345	6 756	6 812
Thorn Forest	3912	3 591	3 591	3 842	3 925	3 666	3 675

Only the most important types of land cover are shown. A, B, and C refers to the three plans; 1 refers to the first solution; 2 to the second. The Null plan is the one in which no spatial criteria are used. The areas are in sq km.

Even at a fine resolution, what was striking was the extent of spatial similarities between the plans. All plans select a large number of areas in central and south-central México, including the densely populated Transvolcanic Belt. Areas in the Chihuahuan desert just south of southwest Texas also have high priority. By and large, the priority areas identified here coincide with those identified as high to moderate priority obtained from a multi-taxa gap analysis of priority areas for biodiversity conservation that included a diverse array of faunistic (all vertebrates, several buttery and insect genera) and oristic groups (several genera of owering plants, but not *Opuntia*) in México [36]. That gap analysis identified as high, very high, and extremely high priority areas large regions of the northwest (including the states of Sonora and Chihuahua), the Transvolcanic Belt, and the southwest (including the states of Guerrero and Oaxaca). Previous regional studies of cacti, including *Opuntia* species, identified the southern portion of the Chihuahuan desert as a priority area; those results are consistent with the areas selected in this analysis [37], [38]. In this region, the selected *Opuntia* priority areas of this analysis widely overlapped with the earlier Mexican gap analysis [36]. However, there were also large regions that this *Opuntia* study identified as being of high priority but the earlier Mexican gap analyses identified as having low or very low priority. This was the case for the southern portion of the Baja peninsula (including the states of Baja California Sur and the southern part of Baja California). Thus this analysis extends those earlier results, which did not include *Opuntia* species. Finally, regions identified as moderate to high priority in the earlier gap analysis coincided with the priority areas identified here in the northern portion of the Baja peninsula (including the northern part state of Baja California). Areas in the Chihuahuan desert just south of southwest Texas also have high priority.

### Tradeoffs in Conservation Area Networks

Table 2 also provides the tradeoffs involved in attempting to optimize shape and connectivity. Measured by the perimeter-to-area ratio, the null solution is four times worse than the one with the best shape (Plan C1). As Plans A and B show, optimizing for shape automatically led to optimization for connectivity. However, best connectivity was achieved when it was explicitly included as a criterion in the multi-criteria analysis (Plan C1). Over all, Plan C1 performs best because it achieves both the highest shape and connectivity performance, and only at a cost of 15% more in the number of cells prioritized. Given that the nominal priority areas are intended to be managed for continued human use, but with a focus on the protection of *Opuntia* species, this 15% cost is almost certainly not too exorbitant a price to pay to achieve the spatial coherence provided by Plan C1.

The selected priority areas for *Opuntia* species show how connectivity can be best achieved between the priority areas. Since biosphere reserves were included in the analysis, some selected areas can easily be connected by them. For example, in the northern part of the country, the Desierto del Vizcano and Valle de los Cirios are large biosphere reserves located in the middle of the Baja peninsula. These reserves can serve to connect the *Opuntia* priority areas selected in the northern portion in the state of Baja California, with those from the southern portion, including the state of Baja California Sur. *Opuntia* priority areas located in the northwest in the states of Sonora and Sinaloa can be connected by the large remnant fragments of natural habitat still available in the region. In central México, the *Opuntia* priority areas can be connected using the results of a previous connectivity analysis which incorporated the representation of endemic mammals [39]. The priority areas established in that study can be used to connect the *Opuntia* priority areas located in the Mexican plateau, the Transvolcanic Belt, and Oaxaca. However, some *Opuntia* priority areas present a more difficult challenge to achieving connectivity and organization into large prioritized blocks. For example, the isolated *Opuntia* priority areas located in the north, south, and Pacific lowlands will require a different approach. In these cases, there must be further tradeoffs between the total size of the areas prioritized for conservation and connectivity.

### Final Reections

The results of this analysis confirm the hypotheses: (i) adequate *Opuntia* conservation in México would require a much larger area than could reasonably be put under protection without routine human presence and use; (ii) it would require incorporation of a wide variety of criteria partly to make management feasible; and (iii) innovative algorithms computational methods would be needed to find satisfactory solutions that have a significant potential for implementation on the ground. It should be emphasized that, although the purpose of this paper was to identify areas where *Opuntia* species can be found under human use, these are also the areas in which microendemics are present and should deserve special attention in future conservation programs.

As Table 2 shows, achieving spatial coherence in shape and connectivity to facilitate dispersal necessary for range expansion and maintenance of genetic diversity came at a price: more area had to be prioritized. Optimizing shape (Plan A), and giving it the same weight as minimizing the area, required 15% more area than meeting all representation targets without concern for shape. If shape was given a relative weight of only one-third relative to the area (Plan B), the additional area required was only 6%. There was a roughly linear dependence between the relative importance of shape and the additional cost in terms of increased area. However, enhanced connectivity could be achieved without additional cost compared to Plan A, also requiring 15% more area beyond the null model (Plan C). This means that, for this data set, while optimizing both shape and connectivity was individually expensive, there was enough correlation between these two parameters to make their joint optimization no more expensive.

Management for conservation and restoration appears to have been successful in many Mexican biosphere reserves, in which human activities include sustainable production and exploitation of natural resources. As a consequence, biosphere reserves have been more effective in preventing land use and land cover change compared to other formally (decreed) protected and non-protected areas [40], [41]. The results presented here suggest that successful conservation programs should expand even beyond biosphere reserves and include other priority areas into expanded conservation area networks. As this analysis shows, such networks can be particular important for the conservation of widely–dispersed taxa as *Opuntia* which overlap with routine human presence and use. Moreover, in a result that has the same implications as this analysis, the multi-taxa Mexican gap analysis identified a significant proportion of priority areas for biodiversity conservation outside the formally (decreed) protected areas [36]. Thus, conservation area networks with sound management for conservation and restoration in extended blocks of multiple–use priority areas provides a more promising alternative for México than traditional formally protected areas which exclude or allow little human presence and use of resources.

It should be noted that the database used for this project is being continually updated and, during the time while this study was being conducted, nine species of *Opuntia* that are new to México have been discovered and their occurrence points have been added to the database. These species were not included in the analysis because they had too few occurrence points for species distribution models to be constructed–their inclusion would not have affected the results of this analysis. Once there are enough occurrence records for these species, the results here should be updated. There is an ongoing effort in that direction.

Turning to computational issues, before the development of the tabu search methods described in this paper, to the best of our knowledge, tabu search had only once been previously applied to problem of identifying conservation area networks [42] but the examples that were solved, besides being small in size, only involved the accomplishment of biodiversity representation targets and no spatial analysis. They were also not intended as practical policy recommendations. Thus the full power of tabu search to solve complex optimization problems was not exploited. The results reported here suggest that tabu search is one of the most promising methods for solving the hard spatial optimization problems that arise during biodiversity conservation planning. The methods discussed here in detail for the first time were used, along with an earlier version of the ConsNet software package, by Conservation International to select priority areas in the Papua province of Indonesian New Guinea [42], in a recent study of Mexican herpetological biodiversity [43], and is being currently being used to prioritize areas in Colombia (M. C. Londoño, personal communication). However, in only the first of these instances (which remains unpublished) was an extensive spatial optimization attempted.

## Materials and Methods

### Study Region and Species Data

For this analysis, México was divided into 431 913 cells with an average area of 4.64 km_2_ (SD  = 0.0041 km_2_) at a 0.02°×0.02° longitude × latitude resolution. All species' data and environmental layers used to model distributions (see below) were georeferenced or resampled to this resolution. Information on the protected areas of México was obtained from the Mexican National Commission on Protected Areas (www.conanp.gob.mx/sig; last accessed 27 July 2010).

Species' occurrence data, which were necessary to construct the species distribution models, were available for 84 species from a comprehensive set of biological collections (see Acknowledgments). There were 1–541 records available for each species (with an average of 66 records, SD  = 92.08; total number of records  = 4 456). However, sufficient data to attempt model construction were available only for 63 of the species listed in Table 1. Of these, 23 were microendemic, 58 were endemic to México, and three were considered Mega-México species (those with a distribution spread across México and the arid parts of the United States, that is, Mega-México 1 [*Opuntia stricta*] or those with a distribution spread across México and south up to northern Nicaragua, that is, Mega-México 2 [*O. puberula* and *O. decumbes*]; [44]). Two (*O. bravoana* and *O. excelsa*) were included as at–risk species by the Norma Oficial Mexicana 059–ECOL–2001 (NOM), a technical standard issued by the Mexican federal government that specifies the conservation status of species [45].

### Models of Species' Distributions

The species distribution models were constructed from species' occurrence points and environmental layers using a maximum entropy algorithm. The Maxent software package (Version 3.3.4; [34]) was used to construct the models. Maxent has been shown to be robust for modeling species distributions from occurrence (presence–only) records for a large number of taxa [46]. Following published recommendations [34], [47], [48], Maxent was run without the threshold and hinge features and without duplicates so that there was at most one sample per pixel; linear, quadratic, and product features were used. The convergence threshold was set to a conservative 1.0×10_−5_. For the AUC, that is, the area under the receiver operating characteristic (ROC) curve [34], averages over 100 replicate models were computed. For each model the test:training ratio was set to 40∶60 following Phillips and Dudìk [34] which means that models were constructed using 60% of the data and tested with the remaining 40%. No attempt was made to model species with fewer than four records because such models are typically unreliable. Consequently, models could be constructed for 63 species.

Constructing species distribution with <10 records is always open to question. Typically ≥20 records are recommended though, according to a recent study [49], Maxent model performance stabilizes at 10 records and even five records are sometimes regarded as sufficient [50]. However, restricting attention to species with ≥10 records would have removed 32 of the 84 species, rather than 21, among them seven more microendemics (see Table 1) of obvious conservation significance. Consequently, it was decided to model these species but to subject the results to expert scrutiny besides using the tests described below. All these models survived such scrutiny.

Two tests were used to assess model performance: (i) A relatively conservative threshold of 0.8 was used for the AUC [49], [50], [51]. (An optimal model would have an AUC close to 1 while a model that predicted species occurrences at random would have an AUC of 0.5. Only one species [*O. wilcoxii*] had an AUC value between 0.8 and 0.9.); (ii) For eight internal training and test binomial tests performed by Maxent (two each for minimum presence, 10 percentile presence, equal sensitivity and specificity, maximum sensitivity plus specificity), a p-value <0.05 was required. In general the results indicated that though there were few data points from the very north of México, there was no reason to believe that there was a substantial bias against those regions in model results.

The environmental layers used are listed in Table 4. These include four topographical variables (elevation, slope, aspect, and compound topographical index) and 19 bioclimatic variables. The latter were obtained from the WorldClim database (www.worldclim.org; last accessed 28 February 2010;[52]). Elevation data were obtained from the United States Geological Survey's Hydro–1K DEM dataset (http://eros.usgs.gov/#/Find_Data/Products_and_Data_Available/Elevation_Products; last accessed 16 February 2012). Slope, aspect, and the compound topographical index were derived from the DEM using the Spatial Analyst extension of ArcMap 9.3.

**Table 4:** Environmental parameters for species distribution models.

Parameters
Annual Mean Temperature
Mean Diurnal Range
Isothermality
Temperature Seasonality
Maximum Temperature of Warmest Month
Minimum Temperature of Coldest Month
Temperature Annual Range
Mean Temperature of the Wettest Quarter
Mean Temperature of the Driest Quarter
Mean Temperature of the Warmest Quarter
Mean Temperature of the Coldest Quarter Annual Precipitation
Precipitation of Wettest Month
Precipitation of Driest Month
Precipitation Seasonality
Precipitation of Wettest Quarter
Precipitation of Driest Quarter
Precipitation of Warmest Quarter
Precipitation of Coldest Quarter
Elevation
Slope
Aspect
Compound Topographic Index

Temperatures are in °C, precipitation in mm, slope in meters. All with a pixel size of 0.01° (1 kilometer resolution).

The output from these models directly quantifies habitat suitability for a species by computing the relative probability of its presence in each cell of the study area. These probabilities establish the potential distribution of a species (and are sometimes interpreted as providing an approximate ecological niche model [53], [54]). The predicted distribution is obtained using biological information such as dispersal behavior and other constraints including vicariance factors that limit the potential distribution. In this analysis, the refinement process used the Uso del Suelo y Vegetación (USV) map [55], a recent digital vegetation map of México which distinguishes between primary and secondary vegetation. Cells transformed into agrosystems and rural or urban settlements were assumed not to be suitable habitats for endemic and at–risk species and were excluded from the potential distributions. Model output was interpreted as probabilistic expectations for each species in a cell [10], [12]. Under this interpretation, the sum of these expected values across a set of cells provides the expected occurrence value for the species, that is, the expected number of cells in which the species would be found.

### Targets of Representation, Area Constraints, and Spatial Goals

The goal of each *Opuntia* conservation plan was to provide adequate representation of each species within a prioritized set of cells with as little total area as possible, after including all existing protected areas, while achieving spatial coherence through compactness of shape and contiguity (connectivity) of prioritized cells. Following what has become standard practice in systematic conservation planning [1], [2], [5], adequate representation was interpreted quantitatively as a specified number of prioritized cells in which the species must be present.

In general, there is no fully satisfactory biological rationale for choosing these quantitative targets of representation [2], [55], [56]. In some circumstances, population viability analyses [57] may provide guidance but such analyses typically require abundance data over a large number of time steps [58]. Such data were not available for a single *Opuntia* species in México. Consequently, targets were assigned to reect the rarity and endemicity status of the species. First, an arbitrary relatively small but widely used target of 10% of the expected occurrence within cells [12] was assigned to the three non-endemic species (*O. decumbens*, *O. puberula*, and *O. stricta*). Next, the same target was assigned to the most abundant species (*O. cretochaeta*) with an expected total representation of 3 417 475 and a target of 100% was assigned to the least abundant species (*O. chaffeyi*) with an expected total representation of 17 635. For all other species, targets were assigned on a linear scale between 10% and 100% in inverse proportion to their expected representation in the study area. (Preliminary runs with upper targets between 80% and 100% resulted in insignificant improvements with spatial economy–less than 3% of the area selected. Since there was no biological justification for any of these values between 80% and 100% they were not used. However, it was presumed that a target <80% would not be sufficient for the geographically rarest, typically microendemic species.)

Satisfaction of these representation targets was one of two hard constraints on the optimization problem. The other was the inclusion of all existing protected areas (which satisfied the spatial goal of alignment to the extent required in this analysis). Since these are legally protected, they provided a natural foundation from which a conservation plan could be devised. The problem then becomes one of constrained optimization: given these hard constraints (that cannot be violated), the problem is to find solutions that minimize the total area falling under the rubric of a conservation plan while maximizing the compactness of shape and connectivity of the set of selected areas. This constrained optimization problem was solved through the construction of a multi–criteria objective function with tabu search being used to find the best solution.

### Multi-criteria Analysis

Methods of multi–criteria analysis have been systematically developed by the decision theory community since the 1980s [22], [59]–[61] and have been applied to a wide variety of decision problems in conservation biology [11], [20], [62]–[64]. Canonically simple decisions involve a single agent (the decision–maker) and a single criterion, for instance, the number of at–risk species accommodated by a conservation plan. Two more complex decision scenarios involve: (i) multiple agents (or stakeholders) and a single criterion; or (ii) a single agent and multiple criteria, for instance, biodiversity representation, shapes of priority areas, and economic cost. There is a formal (mathematical) isomorphism between these two problems because combining preferences of multiple agents according to a single criterion into an objective function is equivalent to combining the distinct preferences of a single agent using multiple criteria. Nevertheless, there is an important philosophical difference [5], [65]: the case of a single agent and multiple criteria only involves aggregation over a single agent's preferences for various objectives whereas, in the case of multiple agents, the aggregation involves the preferences of different agents which may well not be commensurable in many circumstances.

Thus, whenever possible, it is advisable not to use formal methods for incorporating multi-agent preferences but to try to ensure that a single set of preferences emerges through deliberation between agents. This was the strategy followed here. Thus the multiple-agent, single-criterion and the even more complex multi-agent–multi-criteria decision problems will be ignored even though there are obviously a large number of stakeholders for *Opuntia* conservation in México. It will be presumed that the stakeholders will act jointly to choose between various plans and that any differences between them can be modeled using the weights that are given to various criteria in a multi–criteria analysis. Finally, the decision analysis here will not explicitly incorporate uncertainties (except that about species' distributions which are implicitly incorporated through the use of probabilistic expectations) because these cannot be quantified in the present context and are thus best left for incorporation during the formulation of a management plan after a set of areas have been prioritized.

A wide variety of methods for multi-criteria analyses have been proposed [10], [11], [61] which range from those that are extensions of standard single-criterion decision and economic theories [60], [66] to those which are not consistent with it, for instance, the much-used Analytic Hierarchy Process (AHP) [67]. In particular, the AHP suffers from the problem of rank reversal [22]: the inclusion of a new alternative in an analysis can change the relative ranks of existing alternatives even though nothing about their performance has changed. Thus, though this analysis used the simple and transparent preference elicitation method of the AHP, it used a different aggregation formula [21], [22] to avoid rank reversal and achieve a final ranking of alternatives that is consistent with standard multi–attribute value theory (MAVT) [68]. This methodology was previously used to prioritize areas in a multi–criteria analysis for selecting conservation areas in northern Namibia [20] and Indonesian New Guinea [43]; it has also been incorporated into the MultCSync software package for decision support in conservation planning [69].

As in the AHP, preference elicitation was done on a ratio scale between 0 and 9 through binary comparisons. There were three criteria: (i) area, with the number of cells included in the set of prioritized areas as its measurable attribute; (ii) shape, with the perimeter–to–area ratio as its measure; and (iii) connectivity, with the number of clusters (contiguous groups of prioritized cells) as its measure. Three plans were produced using different preferences for these criteria. In Plan A, only the first two criteria were used and both were given equal weight. Plan B also used only the first two criteria but the ratio between the number of cells and the shape parameter was assumed as 3∶1. In Plan C, all three criteria were used and the number of cells, the shape parameter, and the number of clusters were given weights in the ratios 2∶2∶1.

### Optimization

The constrained optimization problem required the minimization of an objective function constructed using the multi–criteria methods just discussed. There were three such objective functions resulting in Plans A, B, and C. Traditionally, the solution of such problems has been approached using two types of algorithms: exact and heuristic. Exact algorithms, by definition, are guaranteed to produce optimal solutions. However, even without the spatial component, these area prioritization problems are NP–hard [10]: they reduce to the well–studied set cover [70]–[72] and minimal cover [73]–[75] problems that can be exactly solved using branch-and-bound algorithms [12]. However, NP–hardness means that large problems may become computationally intractable though the frequency of such a scenario remains debated [10], [12], [76]. Meanwhile, heuristic algorithms based on selecting cells with rare species first or cells with highest “complementarity” values (a “greedy” algorithm which selects cells with the most under–represented species first) have been shown to be at most marginally sub-optimal while remaining computationally fast and easy to implement [10], [12], [77]–[79]. Consequently, these heuristic algorithms have been much more often used in practice than exact algorithms [2].

Once spatial criteria are included, the situation changes drastically. Existing heuristic algorithms do not produce near-optimal solutions and exact algorithms, even when they exist, become intractable for problems much smaller than the ones solved here. As a result, a fairly recently developed class of metaheuristic algorithms have emerged as a tool of choice for spatial conservation planning. These are algorithms that repeatedly use a set of heuristic rules to explore the search space and escape from local optima. While metaheuristic algorithms are not guaranteed to produce optimal solutions, a large body of work shows that they routinely achieve near–optimality [10].

As noted earlier (in the Introduction), the first metaheuristic algorithm used for spatial conservation planning was simulated annealing [14]. However, existing implementations remain slow and only allow a limited number of criteria to be incorporated even after significant recent improvements [16]. In order to improve performance the ConsNet software package, based on tabu search [17], was developed for spatial biodiversity conservation planning [18], [19]. Besides alignment, shape, and connectivity, which were the relevant criteria for this analysis, ConsNet has inbuilt options to incorporate replication: the number of independent contiguous sets of cells in which a species is represented. It also allows the incorporation of an arbitrary number of user–specified criteria. The objective function to be minimized is created using the multi–criteria method described earlier. This analysis used a new version of ConsNet, which will be publicly released soon.

#### Tabu Search

Tabu search is an iterative protocol that uses dynamic memory structures to navigate the search space [17]. In particular, the search maintains a tabu memory, which is a set of rules that prohibits the search from making certain moves. One common tabu restriction is that the search is not allowed to make moves that would undo a recent move. Each tabu restriction remains in effect for a set number of iterations (the tabu tenure). Adjusting the tabu tenure dynamically [80], [81] can improve performance and prevent the search from getting trapped in a local optimum [18], [19]. At each iteration (starting from a current solution), the search evaluates a set of neighboring solutions (its current neighborhood). In general, each neighbor is a simple modification of the current solution, but more complex transformations may also be used. The search then chooses the best neighboring solution which is not tabu, and this becomes the current solution for the next iteration. After each iteration, the memory structures are updated based on the results and outcome of the previous step.

The tabu search in ConsNet has two new features to improve search performance: rule based objectives (RBO) and dynamic neighborhood selection (DNS) [19]. RBOs use binary comparison operators (rather than traditional numeric scores) to rank solutions and make decisions at each iteration in the search [18]. RBOs enable the search to incorporate precise ordinal rankings and may be more compatible with user preferences in some multi-criteria analyses. DNS is a meta-strategy which manages multiple neighborhoods and attempts to choose the best one for the next iteration in the search. A well constructed strategy can be used to moderate the intensification and diversification of the search, and can allow smaller neighborhoods to be used more effectively, reducing the number of evaluations required during exploration.

This analysis introduced a new DNS strategy that performs “aggressive” spatial reorganizations when the search was not finding improving solutions. This strategy was created by observing that certain sequences of spatial rearrangement neighborhoods could be used to transform a solution rapidly while preserving the spatial characteristics. After a certain number of iterations without finding a new best solution, the aggressive DNS strategy implemented one of four different escape modes. The duration and intensity of the escape maneuvers depended on the problem size, recent search progress, and the performance of the current solution. For instance, the search could explore deleting the smallest clusters and then expanding existing clusters. The changes were temporarily locked in place by the tabu tenure, and the search was forced to explore new configurations. While not all of these changes led to improved solutions, the ability to climb out of local optima led to better solutions over the long run.

#### Initialization Heuristics

A metaheuristic algorithm begins with an incumbent solution that it tries to improve upon. This analysis used six heuristic algorithms (built into ConsNet) to generate potential starting solutions (see Table 5). In all cases the cells corresponding to the existing protected areas were included. These heuristic solutions did not incorporate multiple criteria or optimize a formal objective; they were only a quick approximation for solving the basic set cover problem. Thus, these solutions were not very useful except that they serve as a starting point for a more detailed metaheuristic search.

**Table 5:** Heuristic algorithms used to generate initial solution.

Algorithm	Rules	
1	Select cell with species furthest from target.	
	Ties broken using richness.	
	Ties broken using lexical order.	
2	The same as 1, but ties broken with adjacent cell	
	before lexical order use.	
3	Select cells with rarest species.	
	Ties broken using richness.	
	Ties broken using lexical order.	
4	The same as 3, but ties broken with adjacent cell	
	before lexical order use.	
5	Select cells with rarest species.	
	Ties broken using richness.	
	Repeat until a threshold is met for number of satisfied	
	targets.	
	Select cell with species furthest from target.	
	Ties broken using richness.	
	Repeat until a threshold is met for number of satisfied	
	targets.	
6	The same as 5, but ties broken with adjacent cell	
	before lexical order use.	
	Ties broken using lexical order.	

#### Metaheuristic Search

The optimization procedure for each objective involved multiple steps. During the first step, the search chose an initial solution and ran a prolonged search. This step used the “aggressive” dynamic neighborhood strategy to help improve the spatial characteristics of the solution. Next, it carried out an intense refinement search starting from the best solution discovered in the previous step. This search uses a neighborhood which examined a large number of moves at each iteration to make improvements that may have been missed otherwise. This search ran slower, but was useful for refining high quality solutions. Finally, this entire process was repeated from a different initial solution as a starting point. A comparison of solutions obtained from two different starting points can be used to gauge how well the search was converging to a near optimal solution.

Objective A (Plan A) gave equal weight to two criteria: minimization of the number of cells (0.5) and minimization the shape (0.5)(lower values for shape indicate better compactness). The sub-score for the number of cells was scaled linearly between 133 000 and 200 000 and the shape was scaled linearly between0.01 and 0.45. These upper and lower bounds were determined by examining the heuristic solutions. The initial search started from the best available heuristic solution and ran for 4×10^6^ iterations. This number was chosen because it is about 10n, where n is the number of cells. The solution was called A1. Next, anew search was run using the same protocol but starting from the worst heuristic solution; the resulting solution was called A2. (These solutions/plans will also be referred to as Plans A1 and A2; and similarly for Plans/Objectives B and C.)

Objective B (Plan B) considered the same criteria as A but used different weights: minimize the number of cells (0.75) and improve shape (0.25). The search steps followed the same procedure and two more solutions were generated (B1 and B2).

Objective C (Plan C) considered three criteria: minimization of the number of cells (0.4), minimization of the shape (0.4), and minimization of the number of clusters (0.2). The sub-score for the number of clusters was scaled linearly between 200 and 2000. When the computation of clusters is enabled, the search ran slower, particularly if the solution had poor spatial organization. For large data sets with clustering, starting the search from a solution that has coherent spatial organization can save time. For that reason, initial starting point for these runs was the best solution (scored with objective C) from the possible candidates A1, A2, B1 and B2. The initial search ran for 2×10_6_ iterations (fewer iterations could be used because the starting point was a high quality solution). Next the refinement search was run for 50 000 iterations; the best solution was saved C1. Finally, we ran a new search starting from the worst solution among the candidates A1, A2, B1, and B2: the worst solution was chosen to test whether the search was being confined to a local optimum. While there is no guarantee that this protocol detects all such local optima, past experience with tabu search indicates that almost all of them are identified in almost all problems [18]. Repeating the process above yielded one more solution C2.

#### Computational Effort

The search was conducted on a Intel Core i7 940 CPU with 4GB of RAM allocated to ConsNet. The Java virtual machine was the Java Hotspot 64-Bit Server VM (build 14.0-b16, mixed mode) and the operating system was Windows Vista Ultimate Service Pack 1. Since multiple searches were run concurrently, reported wall clock times are estimated based on benchmarks. For Objectives A and B, 412×10^9^solutions were evaluated in 29.0 hours (3.95×10^6^ evals/s). For objective C, 207×10^9^ alternatives were evaluated in 80.6 hours (688 000 evals/s).
